# Is there a trend in inductive effect for different alkyl groups?†

**DOI:** 10.1039/d5ra03716f

**Published:** 2025-06-25

**Authors:** Mark C. Elliott, Colan E. Hughes, Peter J. Knowles, Benjamin D. Ward

**Affiliations:** a School of Chemistry, Cardiff University Park Place Cardiff CF10 3AT UK elliottmc@cardiff.ac.uk

## Abstract

We find there to be no significant difference between the inductive effects of four representative alkyl groups as determined by Hirshfeld charge analysis. The use of alkyl group electronegativity values shows no meaningful correlation with the electron-withdrawing/donating ability of the alkyl groups. The ^13^C NMR chemical shift diverges significantly from the calculated Hirshfeld charges, and we consider the latter to be a more reliable indicator of charge distribution.

## Abstract 2

We find there to be no significant difference between the inductive effects of four representative alkyl groups as determined by Hirshfeld charge analysis.

## Graphical abstract

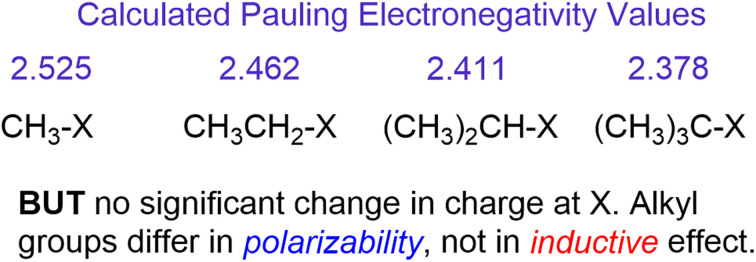

## Introduction

For many years, some organic chemistry textbooks, and the research literature, have given trends in the ability of alkyl groups to exert inductive effects. Ingold reported the inductive electron-releasing effect of alkyl groups as decreasing in the order *t*-Bu > *i*-Pr > Et > Me.^1^ In 1935, Baker and Nathan^2^ gave this same trend. By this time, it was apparently so obvious that no citation was required. In the same work, Baker and Nathan reported the trend for the relative acidity of various alcohols as following the trend:MeOH > EtOH > *i*-PrOH > *t*-BuOH

This was stated to be in agreement with the “generally accepted order of increasing +I effects”. We have been unable to identify a definitive origin for this trend, and speculate that the alcohol acidity trend may even be the origin of the perceived inductive effect trend. Taft and co-workers attempted to separate inductive and polarizability effects involved in the protonation and deprotonation of the above series of alcohols, and derived inductive effect parameters that supported the above trend, and implied a significant difference in the inductive effects.^3^ Some of the assumptions that were made in this work have been challenged.^4^ The alkyl group inductive effect trend was reproduced in Sykes' textbook as late as 1986,^5^ although we have not seen it in other organic chemistry textbooks. In fact, it is now accepted that the trend in relative acidities of the alcohols in aqueous solution are due entirely to solvent effects, with the opposite trend being observed in the gas phase. The gas phase trend can be attributed to differences in polarizability.^6^

We recently presented evidence that alkyl groups are inductively electron-withdrawing relative to hydrogen.^7^ That is, they should be considered to be −I substituents rather than +I. It is important to consider the implications of this work. It is absolutely clear that different alkyl groups can stabilize negative charges to differing extents, and this should be attributed to polarizability rather than to an inductive effect. Similarly, positive charges (ammonium ions, carbocations) are also stabilized to differing extents by various alkyl groups, and this can also be best explained by polarizability or hyperconjugation. In order to place these other effects into proper context, it is necessary to consider whether there is a trend in the underlying inductive effect of different alkyl groups in neutral organic molecules. This, therefore, is the focus of the current work. We will consider the evidence for trends in alkyl group inductive effects, and will examine the data from our previous manuscript in more detail in order to provide much-needed clarity.

## Results and discussion

The electronegativity of alkyl groups can be calculated, with different alkyl groups being considered to have different electronegativities. Based on these data, we would expect *t*-butyl to be less electron-withdrawing than methyl. It is also the case that small structural changes in the carbon skeleton of an organic compound can have significant changes in NMR chemical shifts. Based on these data, we would expect *t*-butyl to be more electron-withdrawing than methyl. We will discuss both aspects in due course, but it is clear from this contradiction that determination of the nature and extent of alkyl group inductive effects is not straightforward. As we noted previously, the IUPAC definition of the inductive effect^8^ includes polarizability^9^ and hyperconjugation^10^ effects, although those are also separately defined.

It is already well established that the above trend for alcohol acidity in the gas phase is a result of the polarizability of alkyl groups.^6^ A *t*-butyl group is more polarizable than a methyl group, and so it is better able to stabilize the negative charge. *t*-Butanol is also more basic than methanol,^11^ since the *t*-butyl group is also better able to stabilize the positive charge. Thus, according to the IUPAC definition, one would have to consider the net electronic effect of a *t*-butyl group (and other alkyl groups) to be either inductively electron-releasing or electron-withdrawing depending on the compound. In both cases, a *t*-butyl group exerts a larger effect than a methyl group, such that it can either be considered to be more electron-withdrawing or more electron-donating than methyl. This position is unsatisfactory from a subject pedagogy perspective.

For this reason, we have continued to restrict our study to the charge distribution in neutral organic molecules to isolate a ‘purely inductive’ effect. For consistency with our previous work,^7^ we used Density-Functional Theory (DFT) calculations with the PBEh1PBE functional^12^ and a flexible orbital basis set (aug-cc-pVTZ^13^), using the Gaussian 09 software.^14^ We will present calculated charges using the Hirshfeld method of charge decomposition analysis.^15^ This method has seen good levels of success in recent years.^16^ As with our previous study, we examined several other methods of charge decomposition analysis (CM5, NBO, Mulliken, QTAIM) and we would reach identical conclusions as discussed in the ESI.†

**Fig. 1:**
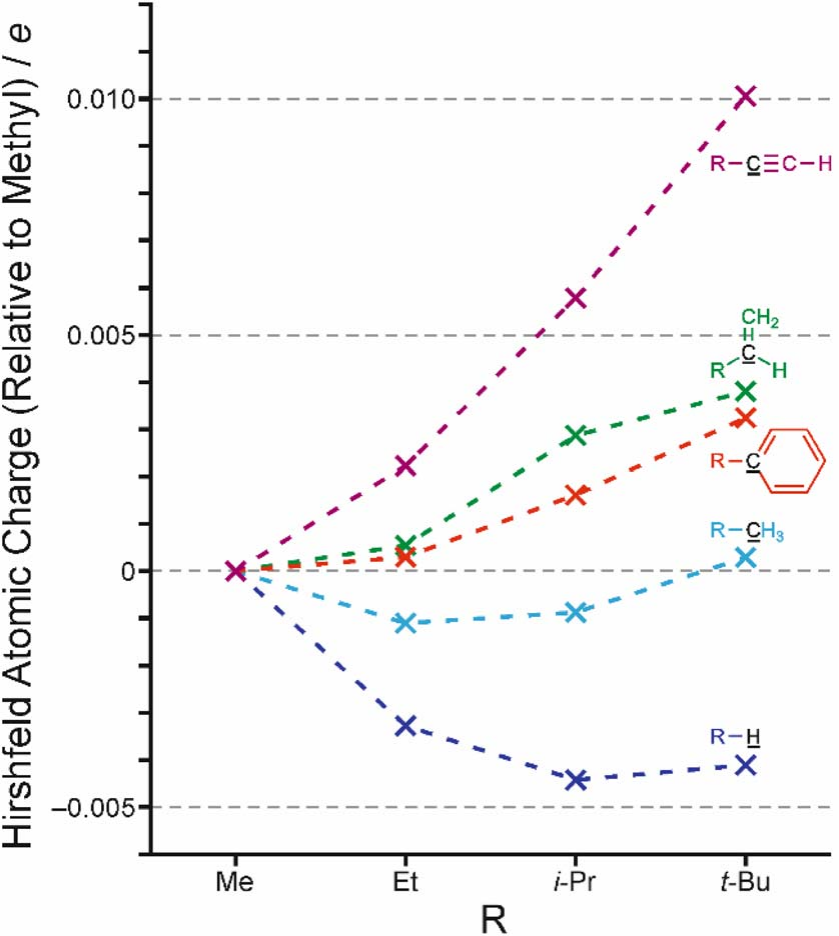
Calculated Hirshfeld charges (*e*) on the black underlined atom relative to Me = 0 in each case.

Fig. 1 is an expansion of a graph given in our previous work, in which the charge at the point of attachment of the alkyl group is now given relative to that for methyl. This allows us to focus on the difference between alkyl group electronic effects.

The first point to note is that the effects are extremely small according to this measure. The second point is that the trends, such as they are, depend on what the alkyl group is attached to. When attached to sp^2^ or sp carbon, the larger alkyl groups are marginally more electron-withdrawing. Based on an inductive effect, in which replacement of three (electron-donating) hydrogen atoms of a methyl group themselves with methyl groups might be expected to result in a net electron-withdrawing effect. What is not clear to us is why only the unsaturated systems are affected in this way.

In reality, the trend for alkyl groups attached to sp hybridized carbon, although the largest, still only spans 0.01*e*. For the other systems, the trends are too small for analysis.

In summary, we find no meaningful difference in the inductive effect across a series of representative alkyl groups. As long ago as 1975, Charton stated that “alkyl groups do not differ significantly in their electrical effects”.^17^

While consideration of calculated atomic charges is probably the most straightforward method for probing electronic effects in organic molecules, other data are available. In light of the current results, we should consider the merit of other potential approaches.

### Calculated alkyl group electronegativity values

It is possible to calculate electronegativity values for alkyl groups. Pauling electronegativity (*χ*) is defined, using the following equation,^18^ in terms of homolytic bond dissociation energies *D*[A–A] and *D*[B–B]. It therefore does not directly relate to the ability of A or B to polarize the A–B bond.*D*[A–B] = ½(*D*[A–A] + *D*[B–B]) + 23(Δ*χ*)^2^

**Fig. 2:**
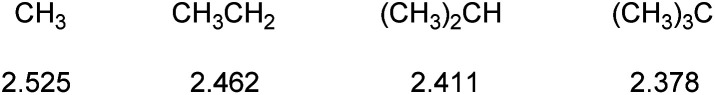
Pauling electronegativity of representative alkyl groups.

The electronegativity of representative alkyl groups, calculated using the above equation, are given in Fig. 2.^19^

**Fig. 3:**
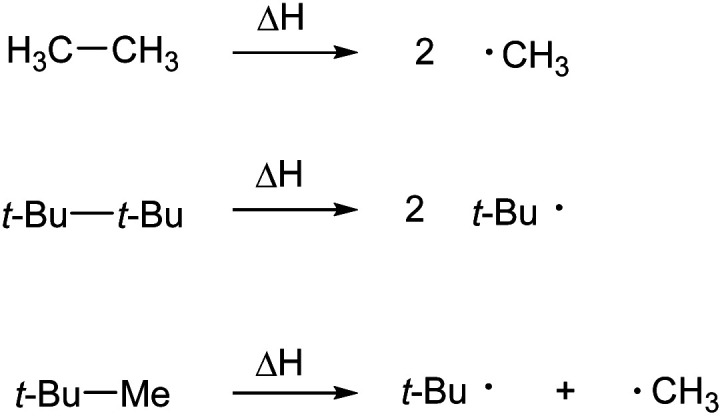
Hypothetical reactions for the calculation of electronegativity of CH_3_ and *t*-Bu.

The relative electronegativities of methyl and *t*-butyl groups can be calculated from the three reactions in Fig. 3, applying a correction for steric effects.^19^

These data agree with the literature alkyl group inductive effect order given in the introduction. However, the second reaction will always be less endothermic than the first, since the *t*-butyl radical is considered to be more stable than the methyl radical. This means that the apparent difference in electronegativity of the methyl and *t*-butyl groups is due to the stabilization of the *t*-butyl radical relative to the methyl radical. While this stabilization has traditionally been presented as a hyperconjugation effect, there is considerable evidence that the amount of stabilization is small and can better be attributed to the relief of steric repulsion in going from the alkane to the corresponding free-radical.^20^ In either case, the difference does not represent a polarization of the C–C bond between the radicals that a difference in electronegativity would imply.

**Fig. 4:**
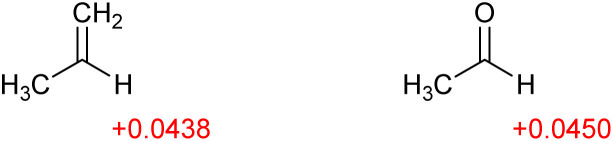
Calculated charges in propene and in acetaldehyde.

A further problem with calculated electronegativity values can be seen in the following example. A vinyl group (2.548) is more electronegative than an acyl group (2.235).^19^ With an electronegative oxygen atom, we would expect an acyl group to exert a stronger electron-withdrawing effect. Calculated Hirshfeld charges (Fig. 4) indicate, in line with our intuitive understanding of electronegativity, that the acyl group exerts a slightly stronger inductive electron-withdrawing effect than prop-2-enyl. The distribution of charge within a molecule would appear to provide a more reliable measure of inductive effects than the electronegativity values.

In summary, we would urge extreme caution in drawing conclusions from calculated electronegativity values as a predictor of alkyl group electronic effects.

### NMR data as evidence of trends in inductive effects of different alkyl groups

**Fig. 5:**
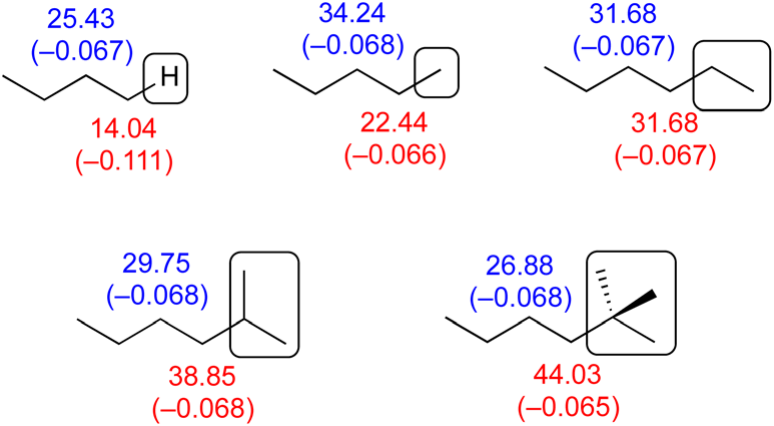
Key ^13^C NMR chemical shifts of selected alkanes (from ref. 21) with Hirshfeld calculated charges shown in parentheses.

It is very tempting to use NMR spectroscopic data as evidence of the differing inductive effects of alkyl groups. The following series of alkanes (Fig. 5), in which a butyl group is attached to hydrogen, methyl, ethyl, isopropyl and *t*-butyl, shows the ^13^C nucleus highlighted in red (α) to be increasingly deshielded in the order H < Me < Et < *i*-Pr < *t*-Bu,^21^ appearing to indicate that these groups are electron-withdrawing in the same order. This, of course, is in contradiction to the trend implied by alkyl group electronegativity values in the previous section. It can also be seen that the ^13^C nucleus highlighted in blue (β) are (except for H) increasingly shielded in the same order.

Salvatella^22^ interprets such results as being consistent with a −I, +R effect, with the −I effect determining the changes in the directly attached nuclei (red) and the +R effect acting on the next atom in the chain (blue).

Calculated Hirshfeld charges are shown in parentheses. As expected, the difference between H and Me is largest, and yet despite the miniscule changes in calculated charge, the difference in chemical shift between Me and *t*-Bu is larger. According to the Hirshfeld charge decomposition analysis, the different alkyl groups show no meaningful difference in inductive effect. One can also recognise a logical problem. Is hexane an ethyl group attached to butyl, or a methyl group attached to pentyl? Each of these compounds can be considered to be a range of different alkyl groups, each having inductive and hyperconjugation effects, and many of these effects will simply cancel out.

**Fig. 6:**
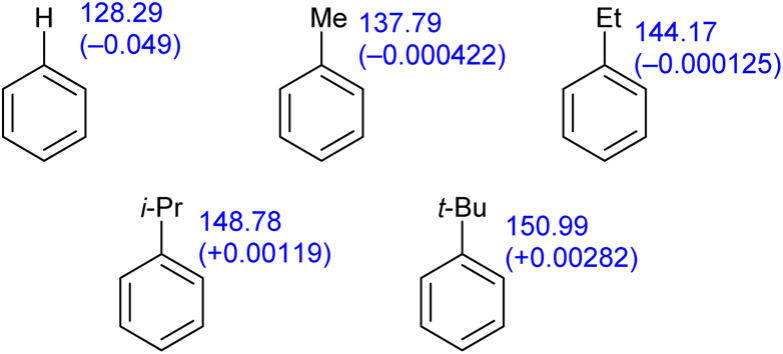
^13^C NMR chemical shifts for the *ipso* carbon of selected alkylbenzenes with Hirshfeld calculated charges shown in parenthesis.

The ^13^C NMR chemical shift for C1 in a range of alkylbenzenes is shown below (Fig. 6). The difference in chemical shift between toluene and *t*-butylbenzene is larger than that between benzene and toluene, which would appear to indicate that the electron-withdrawing ability of a *t*-butyl group relative to hydrogen is more than twice that of a methyl group. The calculated Hirshfeld charges, which we consider to be more reliable, do not support this position.

We see a similar trend in the ^77^Se chemical shifts of dialkylselenides and dialkyldiselenides as shown in Table 1.^23^ There is no perfect system that will allow us to experimentally on a purely inductive effect. However, since carbon and selenium have almost identical electronegativity (which will remove polarizability considerations), and since selenium is in the fourth row of the periodic table, we might expect orbital interactions to be limited. Therefore, despite the reactivity differences between carbon and selenium, it is instructive to explore the ^77^Se data of representative compounds. Again, based only on this data, we might be tempted to conclude that a *t*-butyl group is significantly more electron-withdrawing than a methyl group.

**Table 1:** ^77^Se chemical shifts of representative compounds

	^77^Se shift		^77^Se shift
Me_2_Se	Not reported	Me_2_Se_2_	269
Et_2_Se	239	Et_2_Se_2_	Not reported
*i*-Pr_2_Se	422	*i*-Pr_2_Se_2_	402.5
*t*-Bu_2_Se	600	*t*-Bu_2_Se_2_	488

In summary, while the trend in the ^13^C and ^77^Se chemical shifts could be interpreted as indicating a trend in the relative inductive effects of the alkyl groups, we feel that care should be taken with such an interpretation. The chemical shift is a measure of the response of the electrons to the applied magnetic field rather than a simple measure of local charge density. Calculations of chemical shifts use a range of methods, including machine learning, HOSE codes, and sophisticated DFT methods involving gauge-independent atomic orbitals. Importantly, they do not simply rely on any calculated charge model.^24^ Calculations of chemical shift at the same level of theory are very much in line with the experimental values. That is, a calculation that predicts a large variation in chemical shift also predicts small variation in charge, so that we would not expect good correlation between calculated charge and experimental chemical shift.

## Conclusions

We find, as suggested by Charton half a century ago, that the electronic effects of all alkyl groups are essentially identical, when considered in neutral molecules. The alkyl group electronegativity values are a diversion, since they are not measuring electron-donating/withdrawing effects. Similarly, there is no meaningful correlation between calculated charge and chemical shift. In charged species, polarizability/hyperconjugation effects are expected to dominate, and one is drawn to Ingold's statement that alkyl groups “will exert essentially those polar effects which are impressed on them by the other polar groups present in the molecule”.^25^

In undergraduate organic chemistry textbooks, some trends (alcohol and carboxylic acid acidity; amine basicity) are often attributed to inductive effects, where they might be more accurately explained in terms of polarizability or solvent effects. The inductive effect is generally (and reasonably) presented in the context of electronegative substituents, such that the subtleties of the IUPAC definition are not apparent to the reader. Even with these highly electronegative elements, the inductive effect does not always provide the best explanation for properties such as carboxylic acid acidity.^26^

In our opinion, part of the problem is that there is a disconnect between what most practising organic chemists think of as an inductive effect, and what the strict IUPAC definition (with its inclusion of hyperconjugation and polarizability) states. It is unlikely that common usage of the term ‘inductive effect’ will change. We would strongly advocate teaching carbocation stabilization by alkyl groups only in terms of hyperconjugation (σ conjugation is a better term, to reflect the generality of this mechanism). We suggest that polarizability should be introduced more prominently and more rigorously in organic chemistry textbooks. We suggest that authors be clear whether they are discussing gas phase or solution data, as it is inappropriate to infer substituent effects based on data that contradicts that obtained in the gas phase.

The previous generations of physical organic chemists expended considerable efforts in attempting to partition substituent effects into various components (inductive, resonance, field, polarizability, steric, solvent), and with the advent of modern computational methods it may be that such distinctions are no-longer necessary. The very definition of the inductive effect at present would indicate that such partitioning has not been successful.

## Author contributions

MCE proposed the concept. All authors were involved in the research and discussion of results, and in production of the manuscript.

## Conflicts of interest

There are no conflicts to declare.

## Supplementary Material

RA-015-D5RA03716F-s001

## Data Availability

The computational data supporting the results presented in this article are freely available *via* the Cardiff University Data Catalogue (DOI: https://doi.org/10.17035/cardiff.29092625.v1).
