# Supplementary material for: Is there a trend in inductive effect for different alkyl groups?
Source: RSC Adv. 2025 Jun 25;15(27):21780–4. doi: 10.1039/d5ra03716f (PMC12189267; doi:10.1039/d5ra03716f)
Supplement: RA-015-D5RA03716F-s001 [file RA-015-D5RA03716F-s001.pdf]

Supplementary Information (SI) for RSC Advances.
This journal is © The Royal Society of Chemistry 2025

Is There a Trend in Inductive Effect for Different Alkyl Groups?

Mark C. Elliott,*[a] Colan E. Hughes, [a] Peter J. Knowles [a] and Benjamin D. Ward [a]

[a] School of Chemistry, Cardiff University, Park Place, Cardiff, CF10 3AT

CM5 Charges

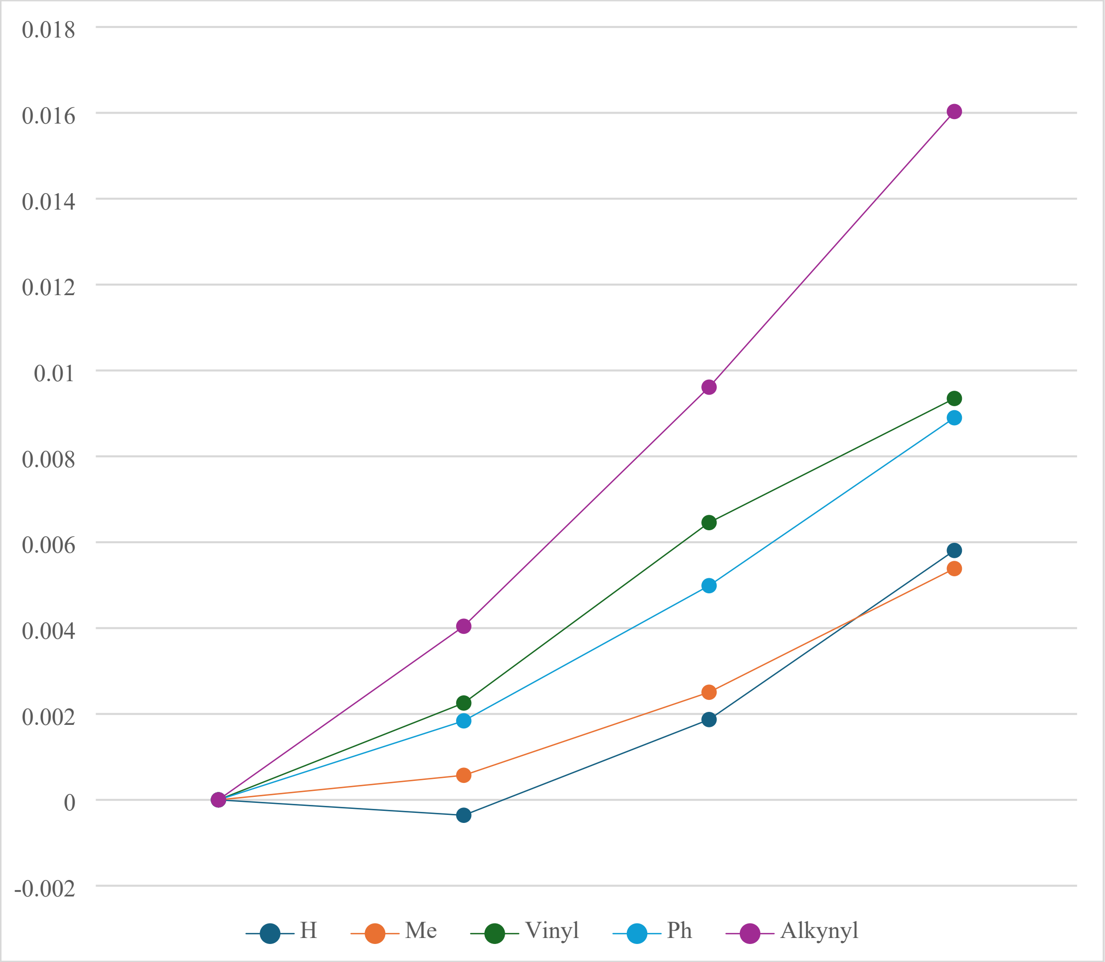

The CM5 charges are perhaps clearer, in suggesting that the additional methyl groups across the series cause an additional, small, inductive electron-withdrawing effect in neutral molecules. This would be consistent with the replacement of each H in a CH_3 group itself with a CH_3 group resulting in a loss of an electron-donating effect that manifests itself therefore as a net electron-withdrawing effect. This is therefore inconsistent with the literature trend for alkyl group inductive effects. As with the Hirshfeld charge analysis, these are miniscule effects, and there is no meaningful difference between the alkyl groups.

NBO Charges

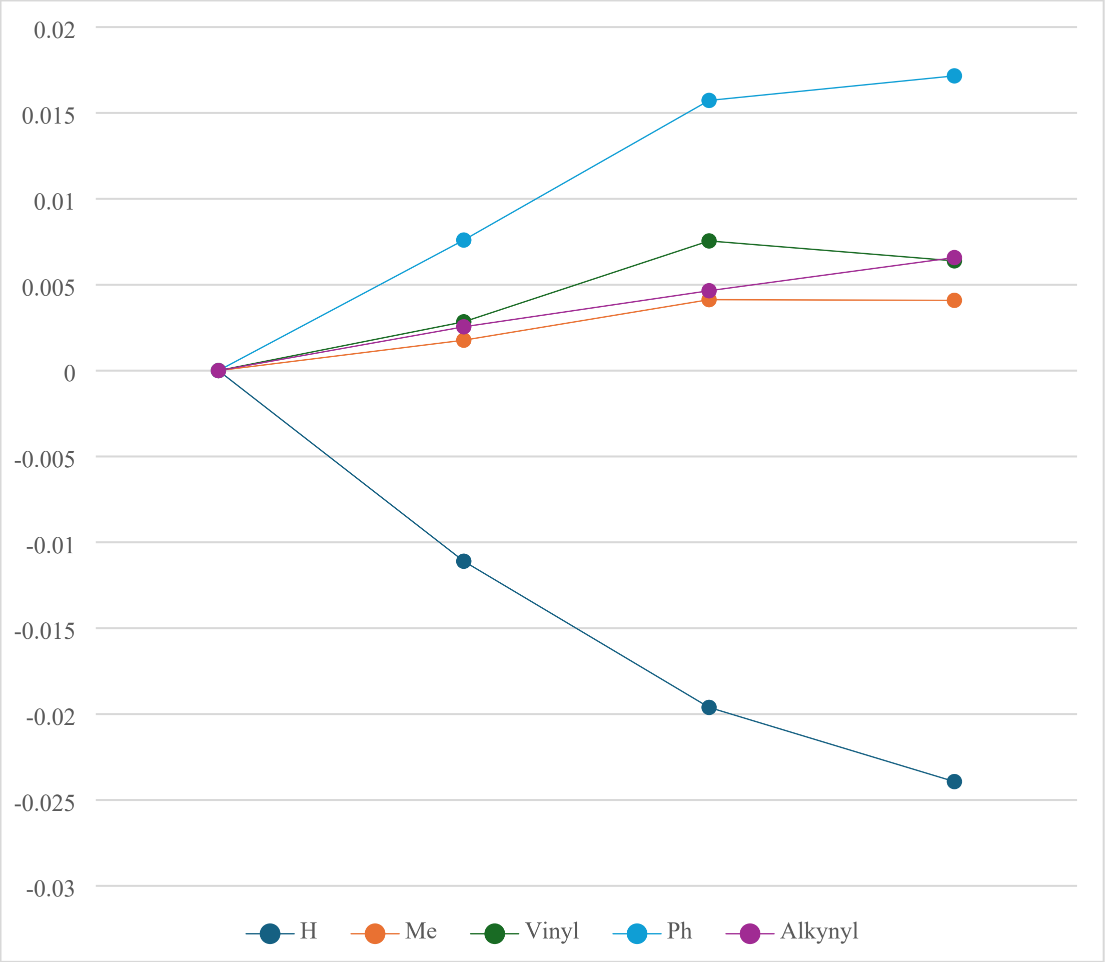

There is a general trend with NBO charges that more C–H hyperconjugation interactions reduces the amount of positive charge on H for each hydrogen. This is not seen in the other charge decomposition models. As with Hirshfeld, the net effects across the alkyl group series are small.

Mulliken Charges

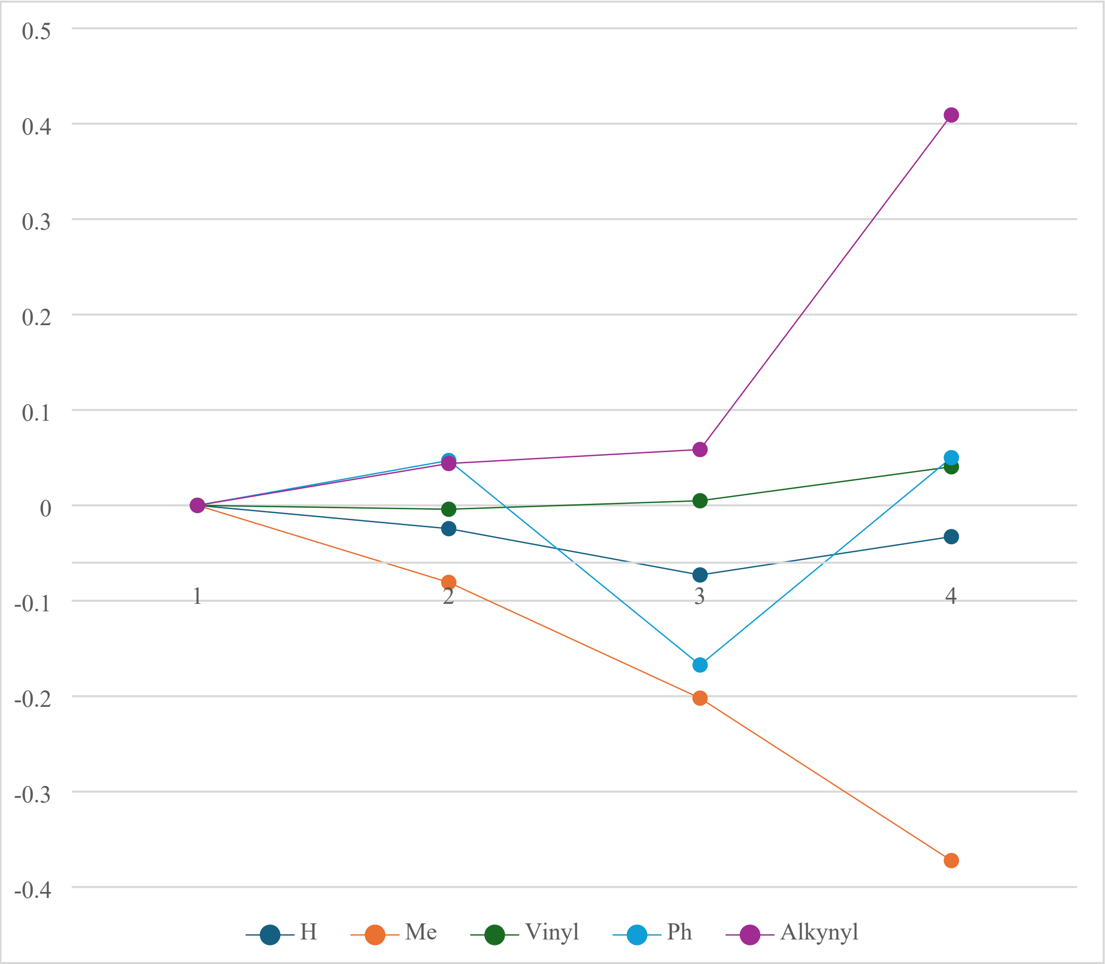

We previously noted issues with Mulliken charges, which are only included here for completeness.

There are many issues here, not least of which is the extent of the effect. A methyl group attached to an alkyne is calculated to have an electron-withdrawing effect of almost 1.2 e compared to hydrogen. Changing this to a *t*-Bu group has an additional electron-withdrawing effect of 0.4 e, with the largest jump between *i*-Pr and *t*-Bu. We can see no logical reason for this change.

QTAIM Charges

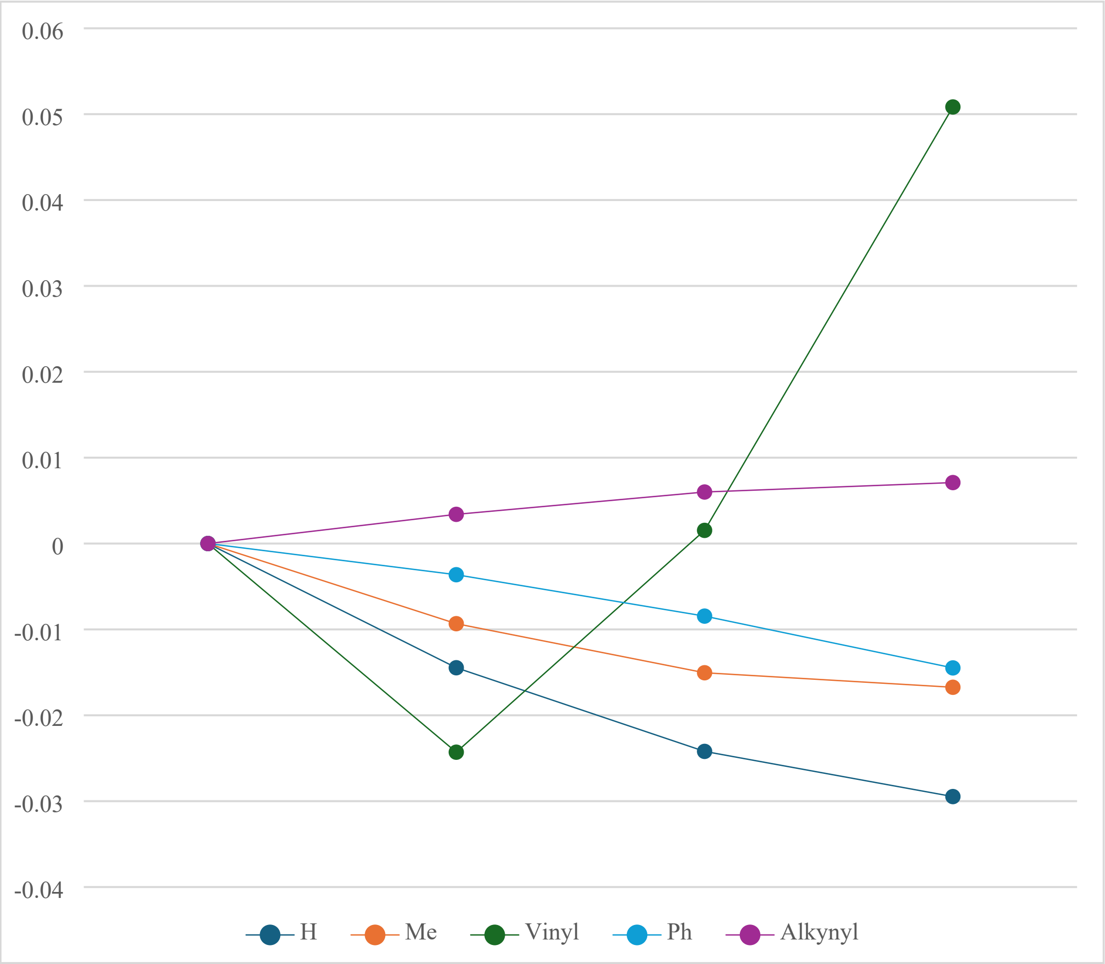

We previously noted some issues with QTAIM charges. Nevertheless, the QTAIM charges for the different alkyl groups show only small differences, and no clear trends.

The only possible exception is alkyl groups attached to an alkene double bond, for which the effect is larger.
